# MALDI-TOF Mass Spectrometry for interrogating ubiquitin enzymes

**DOI:** 10.3389/fmolb.2023.1184934

**Published:** 2023-05-09

**Authors:** Virginia De Cesare

**Affiliations:** MRC Protein Phosphorylation and Ubiquitylation Unit, Sir James Black Centre, School of Life Sciences, University of Dundee, Dundee, United Kingdom

**Keywords:** ubiquitin (Ub), E2 conjugating enzyme, E3 ligase, deubiquinating enzymes, Matrix-assisted laser desorption/ionization time-of-flight Mass-Spectrometry (MALDI-TOF MS), non-lysine ubiquitination, HECT E3 ligase, RBR E3 ligase, RING E3 ligase

## Abstract

The attachment of ubiquitin to a substrate (ubiquitination or ubiquitylation) impacts its lifetime and regulates its function within the cell. Several classes of enzymes oversee the attachment of ubiquitin to the substrate: an E1 activating enzyme that makes ubiquitin chemically susceptible prior to the following stages of conjugation and ligation, respectively mediated by E2 conjugating enzymes (E2s) and E3 ligases (E3s). Around 40 E2s and more than 600 E3s are encoded in the human genome, and their combinatorial and cooperative behaviour dictate the tight specificity necessary for the regulation of thousands of substrates. The removal of ubiquitin is orchestrated by a network of about 100 deubiquitylating enzymes (DUBs). Many cellular processes are tightly controlled by ubiquitylation, which is essential in maintaining cellular homeostasis. Because of the fundamental role(s) of ubiquitylation, there is an interest in better understanding the function and specificity of the ubiquitin machinery. Since 2014, an expanding array of Matrix-assisted laser desorption/ionization time-of-flight (MALDI-TOF) Mass Spectrometry (MS) assays have been developed to systematically characterise the activity of a variety of ubiquitin enzymes *in vitro*. Here we recapitulate how MALDI-TOF MS aided the *in vitro* characterization of ubiquitin enzymes and the discovery of new and unexpected of E2s and DUBs functions. Given the versatility of the MALDI-TOF MS approach, we foreseen the use of this technology to further expand our understanding of ubiquitin and ubiquitin-like enzymes.

## Introduction

Ubiquitin is a small, yet influential protein that regulates a variety of cellular processes (Oh et al., 2018). Ubiquitylation starts with the attachment of a single ubiquitin molecule to a lysine present on the substrate, via isopeptide bond formation (Hershko and Ciechanover, 1998), also referred to as “canonical” ubiquitylation. Besides the canonical addition, ubiquitin can also be connected to protein N-termini (through formation of peptide bond) (Ciechanover and Ben-Saadon, 2004; Tatham et al., 2013; Dittmar and Winklhofer, 2019) or to hydroxyl-containing amino acids and biomolecules, such as serine and threonine (Pao et al., 2018), ADP-ribose (Zhu et al., 2022), glucosaccharides (Kelsall et al., 2022) or the bacterial lipopolysaccharide (LPS) (Otten et al., 2021) via ester bond formation. Recently, phospholipid phosphatidylethanolamine (PE) was also found to be targeted by ubiquitin in eukaryotic cells and baculoviruses (Sakamaki et al., 2022). Besides the attachment to a substrate, ubiquitin itself possesses seven lysine (K) residues (K6, K11, K27, K29, K33, K48, K63) and a starting methionine which can be ubiquitinated, to initiate isopeptide-and peptide-linked ubiquitin chains (Akutsu et al., 2016). Polyubiquitin chains can be either linear or branched and further modified by other ubiquitin post-translational modifications, such as phosphorylation and acetylation (Ikeda and Dikic, 2008; Ohtake et al., 2015; French et al., 2021). The layering and interactions of ubiquitin structural topologies and other post-translational modifications dictates a variety of signals with separate cellular outcomes, referred to as the “ubiquitin code” (Komander and Rape, 2012). The assembly of ubiquitin chains is mediated by a cascade of highly linkage-specific ubiquitin enzymes, E2s and E3s, also known as the “writers” of the ubiquitin code (Zheng and Shabek, 2017). Ubiquitin chains of diverse linkages are recognized and hydrolysed by a dedicated class of enzymes, known as deubiquitylating enzymes (DUBs), which play the role of the “erasers” (Lange et al., 2022). Because of the role of ubiquitination in human health and diseases, there is an ever-expanding interest in how to better characterize the activity of ubiquitin enzymes and how to manipulate them for pharmacological purposes. Both basic research and the drug discovery process benefit of straight forward, easy-to-use, and high-throughput tools to interrogate the activity of ubiquitin enzymes *in vitro*. Here we discuss the use of MALDI-TOF MS-based platforms that allow to gauge ubiquitin enzyme activities, substrate preferences and cooperative behaviour. In perspective, such technology is promising and well positioned to further stretch and expand our understanding of ubiquitin and ubiquitin-like enzymes.

## MALDI TOF MS for interrogating E2 conjugating enzymes and E3 ligases

The ubiquitin enzymatic cascade resembles a pyramid with increasing specificity for the substrate from the top to the bottom. The E1 activating enzyme renders ubiquitin chemically susceptible for conjugation before passing it to an E2 conjugating enzyme (E2) which in turn will collaborate with a specific E3 ligase (E3) to achieve the final task of attaching ubiquitin to a specific substrate (Scheffner et al., 1995; Morreale and Walden, 2016; Zheng and Shabek, 2017). E3 are ultimately responsible for substrate specificity and their sheer number is indicative of the extent of ubiquination that might occur within the cell and alter metabolic processes. Ubiquitin ligases are divided into three families according to the presence of specific domains: Really Interesting New Gene (RING), homologous with E6-associated protein C-terminus, (HECT), and RING-Between-RING (RBR) (Zheng and Shabek, 2017; Harper and Schulman, 2021). RING-type ligases work as scaffolds between the E2 and the target protein substrate, thus allowing the ubiquitylation of the substrate (Metzger et al., 2014). HECT-type ligases possess an active cysteine that forms a covalent thioester bond with ubiquitin before its final transfer to the substrate (Metzger et al., 2012; Scheffner and Kumar, 2014). RBR-type ligases retain both the RING and HECT domains a hybrid RING/HECT-like mechanism of action (Smit and Sixma, 2014; Spratt et al., 2014). A distinct “RING–Cys–relay” (RCR) catalytic mechanism was recently identified in the human E3 ligase MYCBP2, which present a RING domain and two catalytic cysteines that mediate the ultimate attachment of ubiquitin to threonine (Pao et al., 2018; Mabbitt et al., 2020). Finally, a new RING-independent, NFX1-type zinc finger—mediated mechanism was identify in the E3 ligase RNF213 (Otten et al., 2021).

**FIGURE 1:**
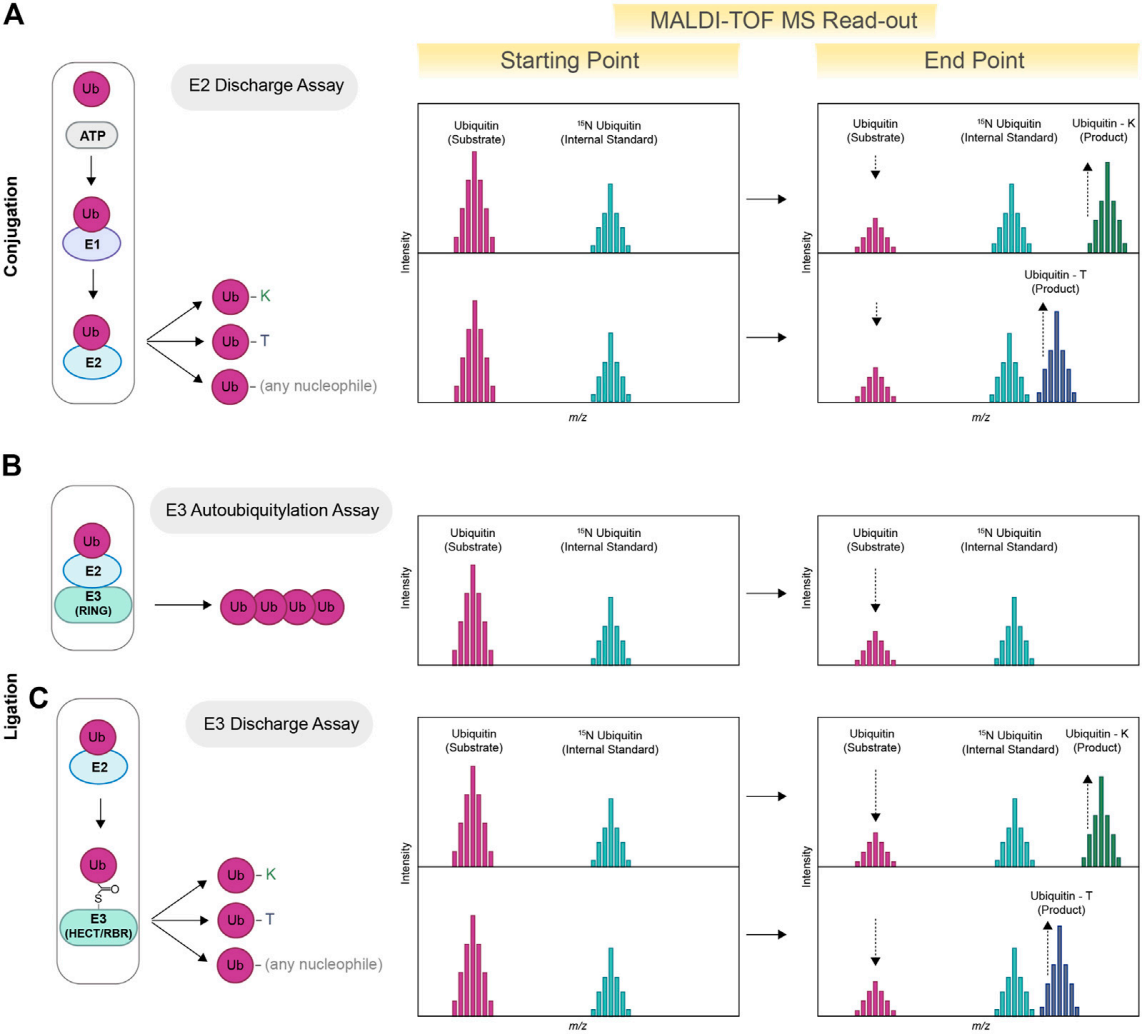
Schematic view of MALDI-TOF MS-based assay for E2s and E3s enzymes. MALDI-TOF MS can be used for determining the specificity of E2s toward specific nucleophiles. Ubiquitin (substrate), ATP/MgCl_2_, E1 and E2 are incubated in presence of excess amount of a specific nucleophile (lysine, threonine or any other nucleophile). Ubiquitin–lysine (ubiquitin-K) and/or ubiquitin-threonine (ubiquitin-T) products are subsequently detected via MALDI-TOF MS (E2 Discharge Assay). Quantification is achieved using heavy-labelled ubiquitin as internal standard (^15^N ubiquitin) **(A)**. E2s paired with compatible E3s will promote the formation of ubiquitin chains, therefore reducing the initial pool of free ubiquitin (E3 Autoubiquitylation Assay). The reduction of free ubiquitin is detected via MALDI-TOF MS and allows for the identification of E2/E3 active pairs **(B)**. HECT and RBR discharge activity is detected via the formation of Ubiquitin-K products or other non-canonical derivatives, for example, Ub-T**(C)** (E3 Discharge Assay).

E2s and E3s act cooperatively to connect ubiquitin molecules either as attached or free polyubiquitin chains. The majority of E2s and E3s link ubiquitin to a lysine residue present on the substrate. E2s that work in tandem with RING E3 ligases have the intrinsic ability to directly discharge ubiquitin onto free lysine provided in excess in the reaction solution, also known as nucleophile reactivity assay (Wenzel et al., 2011). Ubiquitin-lysine adducts formed as result of E2 discharge activity can be easily detected via MALDI-TOF MS (see Figure 1A), thus allowing high-throughput screening aimed to identify and characterize E2 inhibitors and/or activators (Traynor et al., 2022). The ability of MALDI-TOF MS to clearly visualize and quantify a variety of ubiquitin adducts also paved the way for the identification of E2s with non-canonical activities (see Figure 1A) (Abdul Rehman et al., 2023). In particular, it allowed for the discovery of the non-canonical activities of UBE2Q1 and UBE2Q2, recently identified as E2s able to discharge on serine and threonine residues, as well as on other hydroxyl-containing molecules (Abdul Rehman et al., 2023). Similarly, UBE2J2, an E2 conjugating enzyme previously known as able to ubiquitylate residues other than lysine (Wang et al., 2009), was further characterized as a serine and cysteine specific E2. The MALDI-TOF-MS-based discharge assay further allowed for the identification of key catalytic determinants that permit the interaction between ubiquitin and the E2s non-canonical substrates (Abdul Rehman et al., 2023).

The combined action of an E2/E3 active pair ultimately results in a reduction of the initial free ubiquitin pool that can be quantified using MALDI-TOF MS (See Figure 1B): such an approach has been previously employed to identify optimal E2/E3 combinations and to perform high throughput screening for HECT, RBR and RING E3 ligases (De Cesare et al., 2018). HECT and RBR E3 ligases receive ubiquitin from the E2 conjugating enzymes UBE2L3, an HECT-RBR-specific E2 that lacks intrinsic, E3-independent reactivity toward lysine residues (Wenzel et al., 2011). The ability to discharge on lysine—and the consequent formation of ubiquitin-lysine adducts (Ub-K)—relies exclusively on the activity of a cognate HECT or RBR E3 ligase. In this instance, a lysine discharge method can also be used for testing the activity of RBR and HECT E3 ligases of interest (See Figure 1C). For example, MALDI-TOF MS has been employed for the characterization of activators of Parkin (Traynor et al., 2022), an RBR E3 ligase whose loss of function is linked to the development of sporadic Parkinson’s Disease (Kitada et al., 1998). Identification of small molecules able to reinstate Parkin activity represents a potential drug treatment strategy (Miller and Muqit, 2019). Parkin is normally localized in the cytoplasm and maintained in an inactive state. Upon mitochondrial damage, Parkin activity is released by PINK1-mediated phosphorylation (p-Parkin) on serine 65 (S65) or by interaction with phosphorylated ubiquitin (p-Ub) (Narendra et al., 2008; Matsuda et al., 2010; Koyano et al., 2014), which functions as an allosteric Parkin modulator. MALDI-TOF MS has been successfully applied to fully characterise and quantify the extent of Parkin activation in presence of different amounts of p-Parkin (Traynor et al., 2022), to perform primary High throughput screening (HTS) aimed at the identification of new Parkin activators and to the validation of small molecules previously patented as potential Parkin activators. Notably, these assays do not require the development of specific antibodies and/or fluorescent probes. Several structural studies have identified point mutations that release Parkin from in its autoinhibitory conformation and rescue defects in p-Ub binding as well as S65 phosphorylation (Trempe et al., 2013; Wauer and Komander, 2013; Kumar et al., 2015). MALDI-TOF MS also allowed for the highly accurate quantification of the effects of point mutations on Parkin activity, thus providing a precious tool for validating protein structures and/or *in silico* models.

A recent stream of literature identified several E3 ligases that discharge ubiquitin onto non-canonical residues such as serine and threonine as well as onto other biomolecules, including but not limited to sugars and lipids (non-canonical ubiquitylation) (Dikic and Schulman, 2022; Squair and Virdee, 2022). Notably, MALDI-TOF MS can be easily adapted to identify E3 ligases with non-canonical activity in *in vitro* conditions (Kelsall et al., 2022; Wang et al., 2023) (as for discharge on threonine residues) or upon affinity purification steps thanks to a rather high level of tolerance to contaminants (See Figure 1C).

## MALDI-TOF MS for deciphering DUBs activity

**FIGURE 2:**
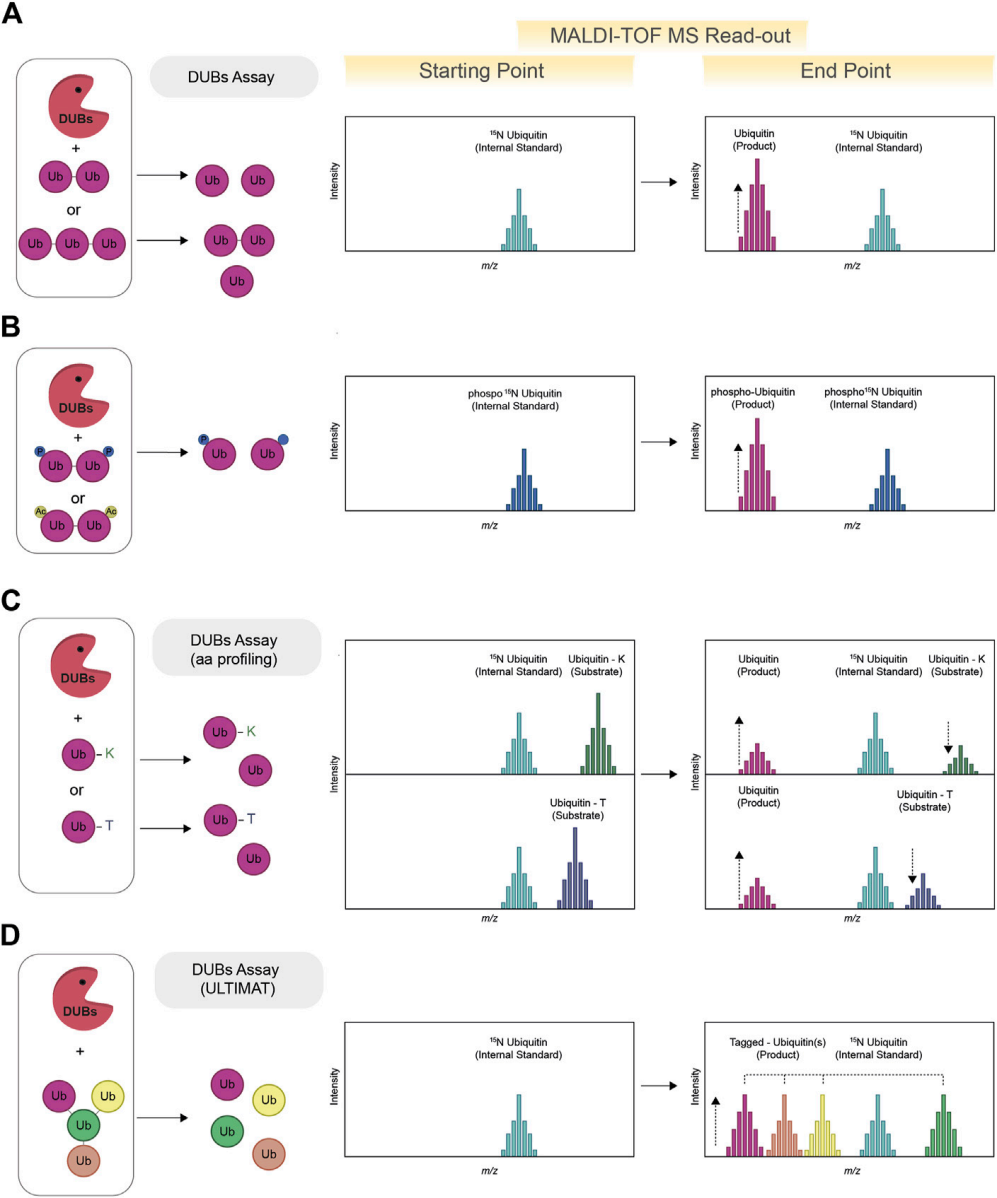
Schematic view of MALDI-TOF MS-based assay for deubiquitylating enzymes (DUBs). The MALDI-TOF MS DUBs assay **(A)** requires the use of ubiquitin dimers (or trimers, tetramers, etc.) as substrates. The formation of ubiquitin as product of the reaction indicates DUBs activity. Quantification and normalization of data points is achieved using ^15^N ubiquitin as internal standard. The activity of DUBs against phosphorylated and or acetylated ubiquitin substrates can also be tested via MALDI-TOF MS by adopting the use of specific internal standards (for example, phosphorylated ^15^N ubiquitin) **(B)**. To determine the ability of DUBs to remove either canonical or non-canonical ubiquitylation, chemoenzymatically synthesized ubiquitinated lysine and threonine are used as model substrates (ubiquitin-K and ubiquitin-T) (aa profiling). The contemporaneous formation of free ubiquitin (product) and reduction of the substrate signal indicated DUBs activity **(C)**. The DUBs mediated cleavage of ubiquitin chains with branching points can be investigated with the use of Ubiquitin Linkage Target Identification by Mass-Tagging (ULTIMAT DUBs Assay) technology. Each ubiquitin moiety of the ULTIMAT substrate is characterized by a slightly different molecular weight that can be detected via MALDI-TOF MS **(D)** thus enabling identification and quantification of the exact linkage cleaved relative to the internal standard (^15^N ubiquitin).

Deubiquitinating enzymes (DUBs) oversee the removal ubiquitin from their substrate and recycle ubiquitin. The human genome encodes for approximately 100 DUBs, divided into 7 families (Clague et al., 2019): Ub C-terminal hydrolases (UCHs), Ub-specific proteases (USPs), Machado-Josephin domain proteases (MJDs), ovarian tumour proteases (OTUs), motif interacting with Ub-containing novel DUB family (MINDY), and zinc-finger-containing Ub peptidase (ZUP1) and the Jab1/Mov34/MPN+ protease (JAMM) family (Lange et al., 2022). Despite sharing the same catalytic activity, DUBs vary in molecular size, structure, and domain architecture, which can also confer specificity toward ubiquitin chain architecture and linkage type. In fact, ubiquitin chains linked via different lysine residues, despite being chemically identical, are considerably different from a structural point of view. MALDI-TOF MS has been extensively used for the characterization of DUB activity toward a variety of ubiquitin substrates, including different chains architecture (linear and branched) (Lange et al., 2023), length (dimer, trimers, tetramer, etc.) (De Cesare et al., 2020; Armstrong et al., 2021), substrates mimicking lysine and threonine ubiquitylation (De Cesare et al., 2021) and ubiquitin substrates further post-translationally modified by phosphorylation (Huguenin-Dezot et al., 2016). MALDI-TOF MS-based assays for determining the activity and specificity of DUBs rely on the detection of free ubiquitin (Ritorto et al., 2014; De Cesare and Davies, 2023). Specifically, the cleavage of ubiquitin dimers results in two mono-ubiquitin molecules: the ubiquitin signal is normalized and quantified based on the internal standard signal, commonly represented by ^15^N ubiquitin (See Figure 2A). While most DUBs are sufficiently active toward ubiquitin dimers, some DUBs cleave poly-ubiquitin chains at accelerated rates compared to ubiquitin dimers, suggestive of an avidity effect or the presence of additional ubiquitin-binding pockets. The cleavage of longer ubiquitin chains will produce the contemporaneous presence of mono-ubiquitin and other ubiquitin chains (i.e., dimer, trimers). The contemporaneous presence of different substrate only marginally complicates the MALDI-TOF MS readout and can be model by following the cleavage reaction *in vitro* over time. For example, PLpro, the SARS-CoV-2 protein with deubiquitylating and deISGylating activities, has proven to be remarkedly more active toward K63 ubiquitin trimers compared to K63 ubiquitin dimers (Armstrong et al., 2021). MALDI-TOF MS allowed for the use of K63 ubiquitin dimers as PLpro physiological substrate in high-throughput screening of 1971 approved clinical compounds and to identify five compounds that inhibited PLpro with IC50s in the low micromolar range (Armstrong et al., 2021).

Ubiquitin can also be targeted by other post-translational modifications such as phosphorylation and acetylation. Phosphorylated ubiquitin is known to activate the E3 ligase Parkin, raising the question of whether phosphorylation could also affect the removal of ubiquitin by impacting the activity of DUBs. Using MALDI-TOF MS, DUBs activity was profiled against different ubiquitin dimers (M1, K6, K11, K48, K63 phosphorylated at three serine (S) residues (S20, S57, and S65) via genetic code expansion (Huguenin-Dezot et al., 2016). The use of phosphorylated ^15^N ubiquitin as internal standard allowed to identify DUBs that were either activated or repressed by the presence of phosphate groups on ubiquitin dimers (See Figure 2B). For many DUBs tested, phosphorylation was found to inhibit the cleavage of all the ubiquitin linkage isomers tested, with S65 phosphorylation leading to the greatest inhibition of DUB activity in most cases. Interestingly, ubiquitin phosphorylation was not found to have a strong effect on ubiquitin chain formation when a comprehensive subset of E2s was tested via SDS-page, therefore suggesting that phosphorylation may regulate the levels of ubiquitin chains primarily through modulating DUBs activity (Huguenin-Dezot et al., 2016).

Ubiquitylation is mostly considered as a lysine-specific post-translational modification, however ubiquitylation of other amino acids, like serine, threonine and other hydroxyl containing biomolecules is an emerging area of interest in the ubiquitin field (Squair and Virdee, 2022). Ubiquitylation mediated by hydroxyl groups is based on the formation of ester bonds, a chemically distinct and more labile bond compared to the isopeptide one. To study the ability of DUBs to remove either canonical or non-canonical ubiquitylation, chemoenzymatically synthesized ubiquitinated lysine and threonine were produced as model substrates (De Cesare et al., 2021) (see Figure 2C), approach also known as amino-acid profiling (aa profiling). A panel of 53 recombinant DUBs, belonging to all seven known DUB families, were tested by MALDI-TOF MS for their ability to cleave the ester bond between the ubiquitin C-terminus and threonine hydroxyl group (esterase activity) or to hydrolyse the canonical isopeptide bond (isopeptidase activity.) Interestingly, most DUBs demonstrated dual selectivity, with one relevant exception, represented by JOSD1, a member of the Machado-Joseph disease (MJD) class. JOSD1 was found to possess highly specific ubiquitin esterase activity rivalling the efficiency of the most active isopeptidases. Interestingly JOSD1, was recently identified as potential novel therapy targeted for a specific subset of leukaemias (Yang et al., 2022) and lung adenocarcinoma (Ma et al., 2022). The identification of JOSD1 esterase activity could both support the understanding of JOSD1 biological function, by facilitating the identification of JOSD1 substrates, and to allow for primary HTS aimed to identify new potent and specific JOSD1 inhibitors.

Endogenous ubiquitin chains have been reported as having complex topology, including branching point that can arise from different ubiquitin lysine residues (Swatek et al., 2019). However conventional SDS-page approaches and the canonical MALDI-TOF DUB assay do not provide information on which specific linkage within a ubiquitin chain is being cleaved. To investigate whether some DUBs have specific selectivity toward branched ubiquitin chains, ubiquitin moieties characterized by slightly different molecular weights have been assembled into branched and un-branched ubiquitin substrates (See Figure 2D). This approach, known as Ubiquitin Linkage Target Identification by Mass-Tagging (ULTIMAT DUBs Assay) relies on the detection, via MALDI-TOF MS, of a specific but slightly different mass for each specific component of the branched poly-ubiquitin chains enabling identification and quantification of the exact linkage cleaved relative to the internal standard (^15^N ubiquitin) (Lange et al., 2023). The screening of 53 human DUBs against K48 and K63 branched and un-branched ubiquitin chains led to the identification of DUBs such as MINDYs and ATXN3 as having remarkable specificity toward branched ubiquitin chains. The development of the ULTIMAT DUB assay provides a new, quantitative, and high-throughput method to monitor cleavage of complex ubiquitin substrates and represents an important improvement compared to existing methods that either provide only qualitative dataset or require the prior development of fluorescent tags, which introduce the potential of both steric inhibition and/or fluorescent artefacts.

## Discussion and future perspectives

Ubiquitylation is a key and highly complex post-translational modification that plays a substantial role in the maintenance of cell homeostasis. The relevance of ubiquitylation in cellular context is recapitulated by the large number of enzymes that articulate the primary attachment as well as the building and the removal of ubiquitin moieties from the substrate. In the last decades, the function of many ubiquitin enzymes and their connection to human health and disease has been explored. However, the understanding of this complex post-translational modification is still only marginal and the function of many E2s, E3s and DUBs remain still largely obscure. The *in vitro* reconstitution of the enzymatic activities and regulation of ubiquitin enzymes often paves the way for a better understanding of their cellular function. In this context the MALDI-TOF MS represents a gold standard for the quantification of E2s, E3s and DUBs activities. Among the advantages of MALDI-TOF MS is that it allows for the use of physiological substrates such as ubiquitin dimers, trimers, tetramers and branched ubiquitin chains. The sensitivity of the MS read out permits the straightforward identification of a variety of ubiquitin adducts and therefore facilitates the discovery of unexpected enzymatic activities, i.e., the non-canonical activities of E2 conjugating enzymes. MALDI-TOF-based strategies can also be applied to the quantification of RBR and HECT E3 ligases activities, including non-canonical ones, such as HOIL-1 and MYCBP2. It can also expedite the *in vitro* detection and quantification of ubiquitination activity toward other non-proteinaceous ubiquitin substrates, such as eukaryotic phospholipids, the bacterial lipopolysaccharide (LPS) ubiquitylation and ADP-ribose.

Of the around 100 DUBs identified in the human genome, approximately two-thirds are currently available for *in vitro* curiosity-driven activity-based assays. Current applications of MALDI-TOF MS have focused on ubiquitin-specific enzymes, however the versatility of this technology allows for the detection of ubiquitin-like modifiers such as SUMO, URM1, UFM1 and FAT10. In fact, most ubiquitin-like conjugating enzymes and pathways resemble those involved in ubiquitylation allowing for similar MALDI-TOF MS-based detection strategies. A further niche of investigation is the identification of DUBs able to process ubiquitin-like domains that are integrated within longer substrates, such as in ribosome biogenesis and function (van den Heuvel et al., 2021).

Overall, we anticipate MALDI-TOF MS-based technologies to substantially increase our understanding of the functioning of E2s, E3s ligases and DUBs by providing researchers in the ubiquitin field with highly accurate, quantitative and high-throughput datasets.
